# A reflection on four impactful *Ambio* papers: The biotic perspective: *This article belongs to* Ambio*’s 50th Anniversary Collection. Theme: Climate change impacts*

**DOI:** 10.1007/s13280-020-01442-5

**Published:** 2021-03-01

**Authors:** Anne D. Bjorkman, Angela Wulff

**Affiliations:** 1 Department of Biological and Environmental Sciences, University of Gothenburg, Carl Skottsbergs gata 22B, 413 19 Gothenburg, Sweden; 2 Gothenburg Global Biodiversity Centre, Carl Skottsbergs gata 22B, 413 19 Gothenburg, Sweden

**Keywords:** Alpine areas, Arctic ecology, Climate change, Coral reefs, Hot spots, Temperature

## Abstract

Climate change represents one of the most pressing societal and scientific challenges of our time. While much of the current research on climate change focuses on future prediction, some of the strongest signals of warming can already be seen in Arctic and alpine areas, where temperatures are rising faster than the global average, and in the oceans, where the combination of rising temperatures and acidification due to increased CO_2_ concentrations has had catastrophic consequences for sensitive marine organisms inhabiting coral reefs. The scientific papers highlighted as part of this anniversary issue represent some of the most impactful advances in our understanding of the consequences of anthropogenic climate change. Here, we reflect on the legacy of these papers from the biotic perspective.

## A lasting legacy

A common theme among these four papers is a call to improve monitoring of both abiotic and biotic factors in order to improve our understanding of the consequences of climate change. The past two decades have indeed seen increased efforts to monitor and to experimentally manipulate environmental changes in order to achieve this goal. For example, the Circumpolar Biodiversity Monitoring Program (CBMP) was developed by the Conservation of Arctic Flora and Fauna working group of the Arctic Council to develop monitoring plans and improve the collection and dissemination of monitoring data and outputs across marine, freshwater, terrestrial, and coastal ecosystems in the Arctic. A recent CBMP special issue in this journal detailed recent trends in many of these organisms (Taylor et al. 2020).

In mountain regions, the Global Mountain Biodiversity Assessment (GMBA; https://www.gmba.unibe.ch), a project of the Future Earth programme, was initiated in 2000 to support the conservation and management of mountain biodiversity, including monitoring of climate change effects on plants, animals, and ecosystem services. Similarly, the GLORIA network (GLobal Observation Research Initiative in Alpine Environments; https://gloria.ac.at/home) was established with the goal of monitoring the composition and diversity of mountain summit vegetation globally. A standardized monitoring protocol was developed and implemented in 2001; since then, summit surveys have been conducted at more than 130 locations on 6 continents. The Mountain Invasion Network (MIREN; https://www.mountaininvasions.org) monitors the influx of non-native plant species along roads and trails in more than 20 mountain regions globally.

For two decades, the Global Coral Reef Monitoring Network (GCRMN; https://gcrmn.net/) has been the foundation for reporting on coral reefs. Improved data and operational standards are currently being incorporated and include three Essential Ocean Variables (EOVs), as identified by the Global Ocean Observing System (https://www.goosocean.org/). For coral reefs, three EOVs are identified to describe reef health; hard coral cover and composition, macroalgal canopy cover, and fish diversity and abundance (Obura et al. 2019). In the second half of 2020, the first global report in 12 years will be released. The datasets include > 1 750 000 observations of 23 different variables recorded from more than 100 000 transects.

Biologists have also become “creative” in their efforts to monitor change over time by using historical photographs and other records of species’ occurrences in order to understand how species’ ranges are shifting as warming accelerates (e.g., Steinbauer et al. 2018; Trant et al. 2020). These records were not initially collected with the goal of monitoring in sight but can be used to develop a longer-term view of how biotic and abiotic conditions have changed over time. The Mountain Legacy Project (http://mountainlegacy.ca/), for example, maintains an archive of 120 000 photos of Canada’s mountains taken between 1888 and 1958 that can be used to detect landscape change (e.g., glacier retreat or upward shifts in the treeline; Trant et al. 2020) over the past 150 years. For coral reef ecosystems, this is naturally more challenging since underwater photography and documentation are more recent and data are scarce. However, some knowledge of long-term ecosystem trajectories can be gained from a combination of isotope dating, archeological deposits, and ethnohistoric descriptions (e.g., Kittinger et al. 2011).

A growing number of networks aim to experimentally test the effects of climate change on organisms. For example, the International Tundra Experiment (ITEX; Henry and Molau 1997) network investigates plant responses to experimental warming at sites across the Arctic and alpine tundra. Similarly, the International Drought Experiment, initiated in 2015, aims to understand the impact of drought on plant communities by experimentally excluding rainfall. In recognizing the critical impact of shifting snow regimes (Callaghan et al. 2011), an increasing number of snow manipulation experiments have been implemented across both Arctic and alpine sites (Wipf and Rixen 2010; Cooper 2014), though to our knowledge, these remain individual efforts rather than standardized experiments organized by a network.

Experimental studies in coral reef ecosystems have recently given some cause for optimism. Transplant experiments between sites of different temperature regimes showed both short-term acclimatory and longer-term adaptive acquisition of heat resistance in the tabletop coral *Acropora hyacinthus* (Palumbi et al. 2014). Other experiments have revealed the complexity inherent in coral ecosystems. For example, when removing a corallivorous snail, coral resilience towards a major warming event increased (Shaver et al. 2018). However, interaction effects of ocean acidification and warming could result in even greater sensitivity compared to single-factor effects (Kroeker et al. 2013).

These and other monitoring and experimental efforts have vastly improved our understanding of the effects of climate change on Earth’s biota. An increasing push to make monitoring and experimental data open access and publicly accessible will further increase their value to the scientific community. For example, the open access BioTIME database (Dornelas et al. 2018) contains nearly 9 million records of species composition and abundance data collected over the past century, across taxa, across biomes, and around the world. Such datasets facilitate a detailed, global view of the impacts of climate change, and how they vary across time and space (Dornelas et al. 2014; Blowes et al. 2019).

## Looking back to the future

As scientists, we can learn a great deal by taking a retrospective approach, including analyzing where past predictions have succeeded or failed. The four papers showcased here have held up remarkably well to the test of time, at least in terms of the “big picture” view they presented. Glaciers have continued to melt at unprecedented rates, Arctic snow regimes have been altered by changing temperature and precipitation patterns, coral reef ecosystems have increasingly experienced bleaching events, and Arctic ecosystems have seen shifts in the distributions and abundances of plant and animal species (IPCC 2013).

As predicted by Callaghan et al. (2004), Arctic regions have seen an increase in the abundance of warm-affiliated plant species over time (Elmendorf et al. 2015). However, much is still unknown in terms of the root cause of this change—for example, whether it is due to shifts in abundance of the species already present or an influx of new species from warmer areas to the south. Interestingly, there is little evidence of widespread change in the local-scale diversity of vascular plant communities in the Arctic, at least for those limited locations where monitoring occurs (Elmendorf et al. 2012b). Our understanding of species-level diversity change in nonvascular plants and lichens is even more limited, as these species are particularly difficult to identify and are thus often excluded from monitoring efforts.

Although numerous warming-driven changes have been documented in Arctic ecosystems, many locations demonstrate remarkable resistance to recent climatic changes (Taylor et al. 2020). For example, while on average Arctic regions have seen an increase in shrubs (Elmendorf et al. 2012b) and “shrubification” is extremely evident in some locations (Tape et al. 2012; Myers-Smith et al. 2019), the majority of single-site studies documented no change in shrub abundance over time or in response to experimental warming (Bjorkman et al. 2019). Callaghan et al. (2004) posited that Arctic plant species are more likely to be negatively impacted by competition with more resource-acquisitive southern species that immigrate into Arctic communities than by warming temperatures directly; the seeming resistance of Arctic plant communities documented thus far could be an indication that this assumption is valid, though direct tests of this hypothesis remain extremely rare.

Much like the plants, Arctic terrestrial animals also show a variety of location-specific trends in diversity and abundance. Large, charismatic species such as muskoxen are likely the best monitored animal species, but assessments exist for a range of species, including lemmings, caribou/reindeer, arthropods, and many of the most characteristic Arctic bird species. Of these, none showed evidence of widespread decline across monitoring sites, though a change in species composition was apparent at some low-Arctic locations (Ehrich et al. 2020), likely indicating the slow movement of southern animal species northward. Similarly, although monitoring of invertebrates has occurred at only a few locations, available evidence appears to indicate a mix of trends, with a loss of diversity in some locations and an increase in others (Gillespie et al. 2020a, b).

Callaghan et al.’s (2011) paper predicting changes in Arctic snow regimes also remains remarkably apropos. Arctic biologists have long recognized the importance of snow conditions for Arctic flora and fauna, and increasing evidence suggests that this recognition is not misplaced. Snow conditions influence a multitude of biotic and abiotic factors, including soil temperatures (Zhang 2005), the height of vegetation and its exposure to conditions above the protective snow layer, the timing of the start of the spring growing season (Assmann et al. 2019), and the timing of nest initiation in many Arctic bird species (Liebezeit et al. 2014). Ongoing snow addition and removal experiments at multiple locations across the Arctic will improve our ability to predict how a changing snow regime will influence these processes (Cooper 2014).

The high alpine areas discussed by Haeberli and Beniston (1998) have also experienced rapid biotic change in response to recent climate warming. In contrast to Arctic locations, mountaintops across Europe have seen a rapid increase in the number of plant species that grow there, and this increase has accelerated in concert with accelerating warming (Gottfried et al. 2012; Steinbauer et al. 2018). As in the Arctic, we eventually expect the taller-growing, more acquisitive species from lower elevations to outcompete high-mountain specialist species, possibly leading to local extinction events. However, there is little evidence for this so far, perhaps because the lower-elevation species have not yet reached sufficiently high abundances at higher elevations.

Before the publication by Goreau and Hayes (1994), coral bleaching as a consequence of global warming raised relatively little interest among both researchers and stakeholders. A survey of published papers revealed ca. 30 publications in the preceding 50 years but well over 1500 publications since 1994. The “Hot Spots” map developed by Goreau and Hayes (1994) has proven its use and is still valid. The authors raised awareness and alerted us to the need for immediate action to mitigate the ongoing coral bleaching events at local, regional, and global scales. Global assessments now highlight coral reefs as an indicator system for climate change effects on marine ecosystems, and they have been proposed as a flagship ecosystem for achieving the Paris Agreement (warming < 2 °C; United Nations Climate Change (UNFCC), https://unfccc.int/). However, as shown by Goreau and Hayes (1994), most coral reefs bleach at 1 °C above the average temperature in the warmest month (1-month duration), suggesting that even the relatively modest goal of limiting warming to < 2 °C may not sufficiently protect coral reef ecosystems.

While coral reef conservation achievements lag far behind the goals (e.g., the Convention on Biological Diversity; CBD 2015), some local management actions have proved to be successful despite large-scale analyses that suggested management would not protect local reefs from climate change effects. One such initiative is the removal of coral predators, commonly performed, and now experimentally tested with promising results (Shaver et al. 2018). Although such efforts to maintain and restore coral reefs are valuable, treating the symptoms without treating the disease (global climate change) is not sustainable in the long run.

## Future challenges and directions

Although many unknowns remain, our increasingly improved understanding of how warming directly impacts geophysical features like ice and snow as well as major groups of plant and animal species enables us to expand our focus to understanding the consequences of these changes for ecosystem functioning and services. For example, the changing composition and increasing height of plant communities in the Arctic can influence soil temperatures, which in turn can influence the amount of carbon released into the atmosphere through permafrost thaw and organic matter decomposition (Blok et al. 2016; Bjorkman et al. 2018). Shifts in the vegetation also feed up to higher trophic levels; experimental findings that warming leads to a decline in lichens could portend trouble for reindeer and other animals that rely on lichens for winter forage (Elmendorf et al. 2012a). Warmer spring temperatures and changing snow regimes drive shifts in flowering time that will likely lead to shorter flowering seasons and reduced resource availability for pollinators (Høye et al. 2013; Prevéy et al. 2018). In mountain regions, the changing glacial landscape has myriad downstream consequences for the diversity and functioning of glacier-fed rivers and lakes (Fell et al. 2017).

The years 2014–2017 were record breaking in successive hot years, coinciding with the most severe, widespread, and long-lasting global coral bleaching event recorded (Eakin et al. 2019). Eakin et al. (2019) highlight that heat stress and bleaching both play a role in subsequent disease, which in turn influences mortality. The impacts of coral mortality extend far beyond corals, with significant changes to the fish and invertebrate community that may last decades. About 500 million people depend on coral reefs for food, coastal protection, building materials, and income from tourism, including 30 million people who are totally dependent on coral reefs for their livelihoods or the land they live on (atolls; Wilkinson 2008).

Beyond the myriad consequences of climate change for biodiversity and the survival of species, the scientific, social, and economic value of the Arctic, alpine, and coral reef ecosystems discussed here is indisputable. Arctic permafrost soils contain twice as much carbon as is currently stored in Earth’s atmosphere; thus, the consequences of Arctic permafrost thaw are of global relevance. The total economic value of ecosystem services provided by coral reef ecosystems exceeds $3.4 billion each year for the U.S. alone (fisheries, tourism, and coastal protection; Brander and Beukering 2013). It is our hope that the increasing awareness of the critical ecosystem functions and services that these key ecosystems provide might finally accelerate actions to mitigate the negative effects of climate change.
